# Mitigation and management of adverse events associated with amivantamab therapy

**DOI:** 10.1093/oncolo/oyaf194

**Published:** 2025-07-22

**Authors:** Narjust Florez, Nicole R LeBoeuf, Julia Rotow, Jennifer A Marks, Joshua K Sabari, Oscar Arrieta, Clarissa Baldotto, Rahul Gosain, Dianne Zawisza, Stephanie McDonald, Emanuela Dylgjeri, Paul Cifuentes, Beth McLellan, Natasha B Leighl

**Affiliations:** Dana-Farber Cancer Institute, Boston, MA, USA; Harvard Medical School, Boston, MA, USA; Dana-Farber Cancer Institute, Boston, MA, USA; Brigham and Women’s Hospital, Boston, MA, USA; Dana-Farber Cancer Institute, Boston, MA, USA; Dana-Farber Cancer Institute, Boston, MA, USA; NYU Langone Health, New York, NY, USA; Instituto Nacional de Cancerología, Mexico City, Mexico; Instituto D’or de Pesquisa e Ensino (IDOR), Rio de Janeiro, Brazil; Wilmot Cancer Institute, University of Rochester, Rochester, NY, USA; Princess Margaret Cancer Centre, Toronto, ON, Canada; Dana-Farber Cancer Institute, Boston, MA, USA; Johnson & Johnson, Horsham, PA, USA; Johnson & Johnson, Horsham, PA, USA; Montefiore Medical Center, New York, NY, USA; Princess Margaret Cancer Centre, Toronto, ON, Canada

**Keywords:** non-small cell lung cancer, epidermal growth factor receptor, mesenchymal epithelial transition, adverse drug reaction

## Abstract

Amivantamab is a fully human bispecific epidermal growth factor receptor (EGFR)–directed and mesenchymal epithelial transition (MET) receptor–directed antibody. Intravenous amivantamab is approved and recommended by treatment guidelines as a first-line treatment (1L) in combination with lazertinib, as a second-line treatment (2L) in combination with chemotherapy in adults with advanced or metastatic non-small cell lung cancer (NSCLC) with EGFR exon 19 deletions or exon 21 L858R substitution mutations, and as 2L monotherapy or 1L in combination with chemotherapy in adults with advanced or metastatic NSCLC with exon 20 insertion-mutations. Compared with previous therapies, novel treatments such as amivantamab may be associated with distinct and unique adverse reactions that potentially require optimized prevention and management techniques. Commonly reported adverse reactions associated with amivantamab treatment regimens include cutaneous reactions associated with EGFR inhibition, such as rash, paronychia, and pruritus; those associated with MET inhibition, such as peripheral edema and hypoalbuminemia; and general effects, such as infusion-related reactions. Recommendations are summarized from published guidelines and the authors’ clinical experience for the prevention and management of adverse reactions associated with amivantamab. An understanding of the expected adverse events with amivantamab regimens, along with the range of prophylactic and management options available, may facilitate maintenance of ongoing treatment in patients deriving clinical benefit and improve patient quality of life on therapy.

**Implications for Practice**

While the introduction of targeted therapies, such as amivantamab, has provided substantial improvements in clinical outcomes in *EGFR*-mutated non-small cell lung cancer compared with chemotherapy, they may be associated with distinct adverse reactions that require optimized prevention and management techniques. This article reviews the adverse reactions associated with amivantamab regimens and the authors’ experience in preventing and managing these reactions. Greater understanding of clinician confidence in optimal management of these adverse effects may support improved patient experience and improved clinical outcomes with adherence to therapy.

## Unmet needs in non-small cell lung cancer management

### Background

Advancements in non-small cell lung cancer (NSCLC) management with the addition of immunotherapy and biomarker-guided targeted therapies have led to improved clinical outcomes compared with traditional chemotherapy across a variety of treatment settings.^1-3^ Of the genomic biomarkers currently tested in NSCLC, alterations in the epidermal growth factor receptor (*EGFR*) gene are among the most frequent driver mutations,^4-7^ and are found in approximately 14%-38% of NSCLC globally.^8^ About 85%-90% of these are exon 19 deletions (Ex19del) or exon 21 L858R (L858R) substitution mutations (common or classic *EGFR* mutations [*EGFR*m]); other less commonly occurring mutations include exon 20 insertion (Ex20ins) mutations and the atypical/uncommon activating mutations (eg, G719X, S768I, and others).^6,7^ These observations provide the rationale for EGFR-targeted agents for NSCLC with these activating mutations, and, as a class of therapeutics, may require distinct strategies to optimize adverse effect management.

### EGFR tyrosine kinase inhibitors

The approval of first- and second-generation EGFR tyrosine kinase inhibitors (TKIs), such as gefitinib, erlotinib, and afatinib in the 2000s, resulted in substantial improvements in clinical outcomes over chemotherapy in *EGFR*m NSCLC.^9-12^ Subsequently, the third-generation EGFR-TKI osimertinib was first approved in 2015 as a monotherapy.^13^ In 2024, osimertinib plus chemotherapy and amivantamab plus lazertinib, were approved for 1L *EGFR*m NSCLC^13,14^; however, so far, only amivantamab plus lazertinib has shown a statistically significant benefit in overall survival.^15,16^

First-line treatment with osimertinib versus first-generation EGFR TKIs improved progression-free survival (PFS; 18.9 vs 10.2 months; *P* < .001), overall survival (OS), and central nervous system disease control,^12,17^ while the addition of chemotherapy to first-line osimertinib resulted in significantly improved PFS compared with osimertinib alone (25.5 vs 16.7 months; *P* < .001); however, OS data are immature.^15^ Recent studies have demonstrated the efficacy of osimertinib in a range of NSCLC settings, including as monotherapy in stage 3 *EGFR*m NSCLC following chemoradiotherapy and in the adjuvant setting.^18-20^

Most patients inevitably develop resistance to EGFR TKIs, including osimertinib, within a median of 17-19 months of treatment.^12,15,21^ Mechanisms of resistance to osimertinib include “on-target” mutations of the *EGFR* gene; “off-target” alterations of other molecular pathways, such as mesenchymal epithelial transition *(MET*) amplifications; and histologic transformation.^22^ These mechanisms of resistance provide the rationale for developing agents that target both the EGFR and MET pathways, such as the EGFR-MET bispecific antibody amivantamab.^23^ The combination of amivantamab with lazertinib, a third-generation EGFR TKI with central nervous system activity,^24,25^ showed improved efficacy over osimertinib for first-line treatment of *EGFR*m advanced NSCLC in the MARIPOSA study; a statistically significant and clinically meaningful improvement in median PFS and OS was observed with amivantamab–lazertinib compared with osimertinib (23.7 vs 16.6 months; *P* < .001, and median not reached vs 36.7 months; *P* < .005, respectively).^16,21,26^ On the basis of clinical study results, amivantamab has become one of the current standard-of-care options for first-line treatment of *EGFR*m NSCLC as well as an established treatment in combination with chemotherapy for treatment of acquired resistance to osimertinib.^21,27,28^

### Adverse events associated with EGFR TKIs

EGFR TKIs are associated with various adverse events (AEs), such as skin reactions, diarrhea, and stomatitis,^29-31^ resulting from EGFR blockade, which differ from the AEs associated with traditional chemotherapy (neutropenia, anemia, and thrombocytopenia).^28,32^ EGFR TKI AEs may also affect patients’ quality of life and may interfere with patients’ ability to remain on treatment. Discontinuation rates due to AEs are reported in up to 8% of patients receiving erlotinib, gefitinib, or osimertinib and up to 29% of patients receiving afatinib in clinical trials,^33^ highlighting the need to better identify and manage these AEs.^34,35^ American Society of Clinical Oncology guidelines recommend that clinicians should provide information about AEs when reviewing treatment options with patients and assess patients’ understanding of potential benefits and risks while identifying strategies to prevent and, if necessary, treat AEs that arise.^36^ Nevertheless, findings from physician and patient surveys indicate substantial knowledge gaps about the management of AEs from EGFR-targeted therapies, highlighting the importance of optimal patient education prior to initiating therapy.^37,38^

In this review, the tolerability profile of amivantamab-based regimens and current recommendations to prevent and manage AEs associated with treatment are summarized. This article aims to provide clinicians and patients with resources for shared decision-making and ongoing effective AE management.

## Amivantamab overview

### Mechanism of action

Amivantamab is a fully human EGFR-MET bispecific antibody that binds extracellularly to EGFR and MET to inhibit ligand binding, promote degradation of cell surface receptors, and activate the immune system via Fc-dependent trogocytosis and antibody-dependent cellular cytotoxicity.^39^ Because amivantamab binds extracellularly to EGFR and MET, it inhibits both pathways independent of their intracellular cancer-driving or treatment-acquired mutations.^39^ Additionally, amivantamab’s immune-activating effect distinguishes it from other EGFR inhibitors.^39^

### Amivantamab indications and efficacy

The indications for which amivantamab is US Food and Drug Administration approved,^14^ the corresponding National Comprehensive Cancer Network guideline recommendations,^27^ and the supporting study data are summarized below.

#### First-line treatment combined with lazertinib in NSCLC with EGFR Ex19del or L858R mutations (NCCN Category 1 recommendation)

First-line amivantamab plus lazertinib demonstrated a significantly longer median PFS (23.7 months) versus osimertinib (16.6 months; HR, 0.70; 95% CI: 0.58-0.85; *P* < .001) and OS (median not reached versus 36.7 months, respectively; HR 0.75; 95% CI: 0.61-0.92; *P* < .005) in patients with advanced/metastatic *EGFR*m NSCLC in the MARIPOSA study. Based on an exponential distribution analysis, the median OS benefit is projected to be >1 year.^16,21^

#### Second-line treatment combined with chemotherapy in NSCLC with EGFR Ex19del or L858R mutations (NCCN Category 1 recommendation)

Second-line amivantamab plus chemotherapy provided a significantly longer PFS of 6.3 months compared with 4.2 months with chemotherapy alone (HR 0.48; 95% CI: 0.36-0.64; *P* < .001) and a higher overall response rate (ORR; 64% vs 36%, respectively; *P* < .001) in patients with advanced/metastatic *EGFR*m NSCLC after disease progression on osimertinib in the MARIPOSA-2 study.^28^

#### First-line treatment combined with chemotherapy in NSCLC with EGFR Ex20ins mutations (NCCN Category 1 recommendation)

First-line amivantamab plus platinum-based chemotherapy provided a significantly longer median PFS versus chemotherapy alone (11.4 vs 6.7 months, respectively; HR for disease progression or death, 0.40; 95% CI: 0.30-0.53; *P* < .001) in patients with advanced/unresectable *EGFR* Ex20ins-mutated NSCLC in the phase 3 PAPILLON study.^32^

#### Second-line monotherapy after prior progression on chemotherapy in NSCLC with EGFR Ex20ins mutations (NCCN Category 2A recommendation)

Amivantamab monotherapy demonstrated an ORR of 40% (95% CI: 29-51) and a median PFS of 8.3 months (95% CI: 6.5-10.9) in the open-label CHRYSALIS study.^40^ In comparison, a real-world analysis of patients with *EGFR* Ex20ins-mutated NSCLC demonstrated a 14% ORR and a median PFS of 3.3 months across second-line treatments after platinum chemotherapy.^41^

## EGFR-associated amivantamab AEs

### On-target versus Off-target effects

**Figure 1.**
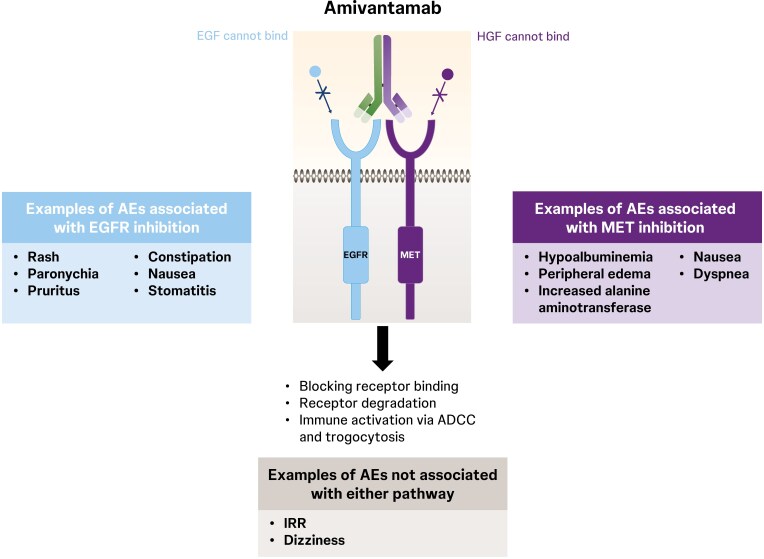
Molecular pathways targeted by amivantamab with associated adverse effect profile.^39,43^ ADCC, antibody-dependent cellular cytotoxicity; AE, adverse event; EGF, epidermal growth factor; EGFR, epidermal growth factor receptor; HGF, hepatocyte growth factor; IRR, infusion-related reaction; MET, mesenchymal epithelial transition.

Targeted cancer treatments are associated with on-target and off-target AEs.^42^ On-target AEs with amivantamab include skin and gastrointestinal reactions associated with EGFR inhibition and peripheral edema, hypoalbuminemia, and transaminase elevation associated with MET inhibition (Figure 1).^29,44-46^ Additionally, IV amivantamab, as with other infused therapies, can be associated with infusion-related reactions (IRRs), with symptoms including chills, nausea, shortness of breath, hypotension, and rash.^47,48^

### Common AEs

Many amivantamab-associated AEs are also shared with other EGFR-targeting therapies, as wild-type EGFR is widely distributed in the skin; thus, dermatologic toxicities are the most common AEs associated with EGFR-targeting monoclonal antibodies and TKIs.^49^

#### Rash

In amivantamab studies, “rash” is used to describe a variety of skin reactions, including acneiform rash, dermatitis, erythema, folliculitis, pustules, maculopapular rash, skin exfoliation, and other skin lesions. The most common specific skin toxicity, often described as acneiform rash, is characterized by papules and pustules with pruritus, pain or burning, and bleeding,^50-53^ and typically presents on the scalp, central face, upper chest, and back. Scalp rash can be challenging to treat and can impact patients’ quality of life.^54-56^ Acneiform eruptions are more common in males and younger patients, likely due to age-related decline in incidences of acne and due to androgen-related increase in EGFR expression.^57^

Rash was the most reported EGFR inhibitor-related AE in clinical trials of amivantamab, although these trials were not optimized to prevent dermatologic toxicities. Any grade rash was reported in 43%-89% of patients across monotherapy and combination therapy studies.^21,28,32,40,58^ The frequency of rash is lower with other EGFR-targeted therapy, with osimertinib associated with the lowest frequency (34%-58%) compared with the earlier generation EGFR TKIs gefitinib (49%-85%), erlotinib (71%-80%), and afatinib (67%-89%).^44^ The anti-EGFR monoclonal antibodies cetuximab and panitumumab, used in colorectal cancer treatment, are associated with rates of any grade rash ≥80%,^59-61^ though less frequently severe. Amivantamab-associated rash was mostly mild to moderate severity, with rates of grade ≥3 rash ranging between 4% and 15% across amivantamab study arms.^21,28,32,40^ Rash generally occurred within 4 months of amivantamab initiation and decreased over time.^62,63^ In the MARIPOSA study, 55% of patients experienced their first onset of rash during the first 4 months of treatment compared with 2% during months 5 through 8, and 1% during months 9 through 12.^62^

#### Paronychia

Paronychia, an inflammatory process involving the soft tissues around the nail,^53,64^ was reported by 37%-68% of patients treated with amivantamab across pivotal trials; most reports were mild to moderate in severity, with the rate of grade ≥3 events ranging from 1%-11%.^21,28,32,40,58^ Paronychia was reported in 22%-35% of patients in osimertinib trials but was not reported as a common AE in trials of gefitinib, erlotinib, or afatinib.^9,12,65^ In the authors’ experience, outside of clinical trials, paronychia is seen more frequently in patients on prolonged rather than shorter courses of EGFR inhibitors.^50,51^

As with rash, paronychia generally occurs within the first 4 months of amivantamab treatment and decreases over time.^62,63^ In the MARIPOSA study, 50% of patients had their first occurrence of paronychia during the initial 4 months of treatment compared with 12% during months 5-8 and 3% during months 9-12.^62^

Although not commonly severe, paronychia may be associated with complications, such as infections and bleeding at the nail beds, and chronic paronychia can be painful and debilitating, even at lower grade manifestations.^51^ In severe cases, periungual pyogenic granulomas can form around the nails. Painful cracks and skin fissures of the hands and feet have also been described.^54^ Other nail changes associated with EGFR inhibitors include mild onycholysis, brittle nails, and slower nail growth.^52^ Notably, MET inhibitor-associated peripheral edema can make the management of paronychia even more challenging; based on the authors’ experience, patients with edema are at high risk of secondary infections in the setting of a chronic skin source of bacteria at the nail.

#### Other EGFR-related AEs

Other EGFR therapies are associated with AEs that have not been reported with amivantamab monotherapy, such as anemia, aminotransferase elevations, neutropenia, thrombocytopenia with gefitinib, and prolonged QT interval with osimertinib.^9,12,15^ When amivantamab is combined with additional drugs in combination regimens, other AEs, as discussed in the following section, may occur.

### Guidelines for managing EGFR-related AEs

Guidelines address the prevention and treatment of AEs associated with EGFR therapies, including those developed by the Multinational Association for Supportive Care in Cancer (MASCC; 2011),^50^ the Oncology Nursing Society (ONS; 2020),^66^ the European Society for Medical Oncology (2021),^51^ a UK multidisciplinary expert panel (2015),^64^ the Taiwanese Dermatological Association (2017),^53^ the Spanish Society for Medical Oncology (2019),^55^ and a Brazilian oncodermatology expert panel (2020).^56^

### Preventive and management strategies

**Figure 2.**
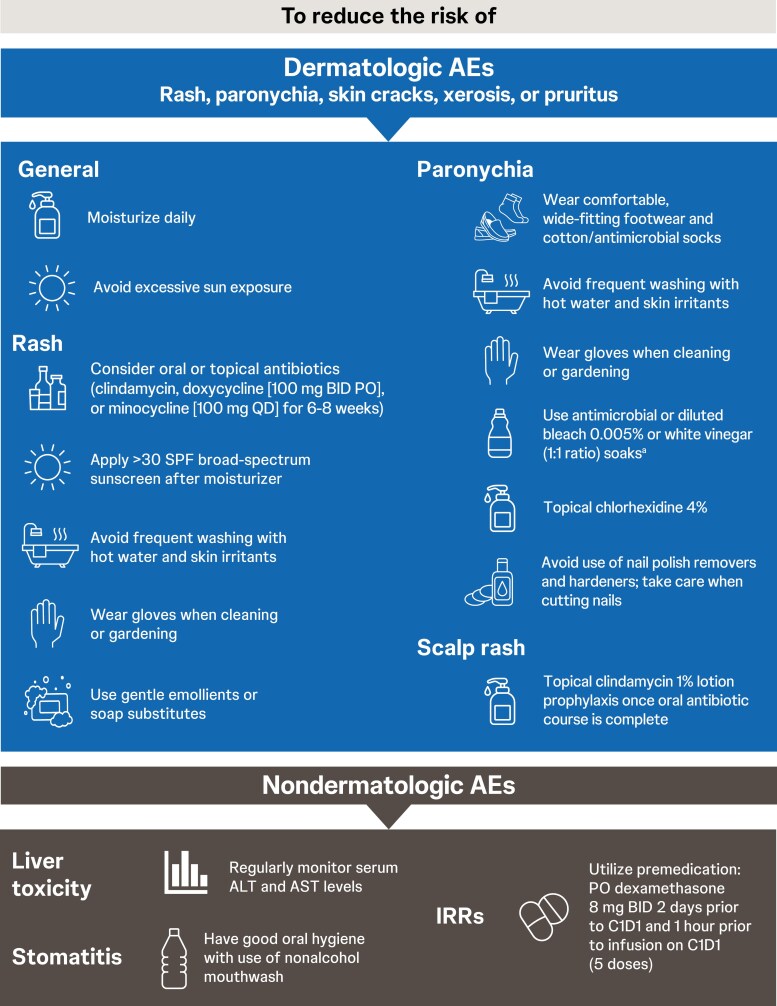
Recommended preventive strategies for reducing the risk of AEs associated with EGFR inhibitors, including amivantamab treatment^43,44,47,48,50-54,56,64,66,68-70. a^Bleach and vinegar should not be mixed. AE, adverse event; ALT, alanine aminotransferase; AST, aspartate aminotransferase; BID, twice daily; EGFR, epidermal growth factor receptor; IRR, infusion-related reaction; PO, orally.

The MASCC Skin Toxicity Group and ONS guidelines highlight the importance of patient education regarding dermatologic toxicities before treatment initiation and strongly encourage preventive measures as well as interdisciplinary collaboration.^50,66,67^ For agents like amivantamab, with a high likelihood of cutaneous AEs, a referral to dermatology or oncodermatology upon treatment initiation may be beneficial. More frequent clinic visits may be needed when AEs are expected. Additionally, patients should receive written information regarding at-home strategies for preventing dermatologic toxicities. Nurses, caregivers, and other support staff also require education to assist patients. Figure 2 summarizes recommended preventive strategies to reduce the risk and/or severity of AEs. In addition to lifestyle strategies, tetracycline antibiotics have been used for the prevention of rash. From the authors’ experience, it is recommended to use prophylactic tetracycline antibiotics; however, some practitioners prefer to wait for the first sign of a rash before considering oral antibiotics, so as not to disrupt the gut microbiome. Zinc deficiency has been suggested to play a role in EGFR activation and expression; thus, zinc supplementation has also been used to ameliorate rash.^71-73^

Despite efforts to prevent dermatologic AEs, they do often occur; patients should be instructed to immediately notify their care team as soon as AEs arise so that treatment or dermatologic encounters can be started promptly. Table 1 summarizes the recommended actions to take if AEs occur with amivantamab treatment. Treatment in practice may vary by region, depending on available resources.

**Table 1.** Recommended actions in response to AEs associated with amivantamab treatment.^5,6,12,19,25,30,38,39,42,46,48-57,59,64,66,68,74-79^

Association	AE Type	CTCAE grade	Management action
*EGFR* inhibition	Rash^a,b,c,d,e,f,g^ Frequency: Ami monotherapy = 86%; Ami + Laz = 62%; Ami + CT = 43%-54%	Grades 1-2	→Continue mild fragrance-free moisturizers twice daily after showering/bathing →Start or increase dose of topical corticosteroids (hydrocortisone cream or ointment 2.5% or equivalent for face, triamcinolone 0.1% or equivalent for body) →Medium (triamcinolone 0.1%) to high potency steroid applied to body BID →Use only a transient (≤ 2 weeks) higher potency steroid for the face with step down to low potency ◦ Triamcinolone 0.1% ointment BID (medium potency) or clobetasol 0.05% ointment (high potency) ◦ Hydrocortisone 2.5% cream or ointment BID (low potency) →Start or increase dose of oral/topical antibiotics (clindamycin lotion, doxycycline 100 mg BID, minocycline 100 mg QD or BID) →Consider topical calcineurin inhibitors (tacrolimus ointment or pimecrolimus cream) for persistent or refractory skin rash with concurrent subspecialty consultation with dermatology if not already consulted
		Grade ≥ 3	→Consider oral isotretinoin if refractory to oral antibiotics and topical regimens (dermatology consult may be warranted) →Perform bacterial culture if secondary infection is suspected (such as presence of pustules or impetiginization) →Grade 3: withhold amivantamab and initiate supportive care management. Upon recovery to grade ≤ 2, resume amivantamab at a reduced dose. If there is no improvement within 2 weeks, permanently discontinue amivantamab →Grade 4: permanently discontinue amivantamab
	Scalp rash^a,b,c,d,e,f,g^ Frequency: Not reported	Grades 1-2	→Clobetasol 0.05% solution as needed →Shampoos containing antifungal agents, coal tar, or salicylic acid for seborrheic dermatitis of the scalp →Topical corticosteroids in oil, solution, or lotion vehicles
	Alopecia^a,b,c,d,e,f,g^ Frequency: Not reported	Any grade	→Topical minoxidil (nonscarring alopecia) →Topical steroids (inflammatory and scarring alopecia)
	Paronychia^a,b,c,e,g^ Frequency: Ami monotherapy = 45%; Ami + Laz = 68%; Ami + CT = 37%-56%	Grade 1	→Continue moisturizing and practicing appropriate hand hygiene →Add skin glue as needed for fissures →Warm water or white/apple cider vinegar soaks (1:1 ratio; soak for 10 minutes 3-4 times per day) →Chlorhexidine soaks →Consider topical corticosteroids, calcineurin inhibitors, or beta-blockers (dermatology consult may be warranted)
		Grade 2	→Consider oral and/or topical antibiotics or antifungals →Consider cryotherapy (podiatry or dermatology referral) →Consider silver nitrate chemical cauterization for hypergranulation or pyogenic granuloma formation (podiatry or dermatology referral)
		Grade ≥ 3	→Consider dermatology/podiatry referral for interventional options (eg, partial nail plate avulsion, silver nitrate chemical cauterization) →Swab for bacterial culture if there is a concern for bacterial superinfection →Consider dose interruptions or reductions
	Cracks/skin fissures/xerosis^a,c^ Frequency: Ami monotherapy = 16%; Ami + Laz = 16%; Ami + CT = < 15%	Grades 1-2	→Continue moisturizing →Occlude with liquid bandage for small fissures →Apply bland ointment (ie, Vaseline or white petrolatum) or urea cream 10%-40% at bedtime and cover with cotton gloves/socks as needed →Consider orthopedic insoles or a soft silicone foam dressing to protect the skin →Use ammonium lactate, urea, or lactic acid creams for scaly areas
		Grade ≥ 3	→Consider topical steroid creams for severe xerosis →Grade 3: withhold amivantamab and initiate supportive care management. Upon recovery to grade ≤ 2, resume amivantamab at a reduced dose. If no improvement within 2 weeks, permanently discontinue amivantamab →Grade 4: permanently discontinue amivantamab
	Pruritus^a,c,d,g^ Frequency: Ami monotherapy = 17%; Ami + Laz = 24%; Ami + CT = 15%	Grade 1	→Continue fragrance-free moisturizers →Apply ice packs as needed →Consider topical pramoxine →Consider topical moderate/high-potency steroids (hydrocortisone 2.5% or equivalent for face, triamcinolone 0.1% or equivalent for body, clobetasol or equivalent for palms/soles/scalp) →Consider doxepin
		Grade 2	“Step-up” therapies as needed: →Moisturizers →Ice packs →Oral antihistamines →Oral cetirizine 10 mg daily or loratadine 10 mg daily →Topical steroids if limited areas of pruritus →Doxepin →Gabapentin or pregabalin titrated to patient symptoms
		Grade ≥ 3	→Consider interrupting treatment until mild/no pruritus symptoms (grade ≤ 2)
*MET* inhibition^h^	Peripheral edema Frequency: Ami monotherapy = 18%; Ami + Laz = 36%; Ami + CT = 30%-32%	Grade 1	→Address comorbid conditions if contributing to edema (eg, volume overload) →Most interventions have limited efficacy against MET inhibitor-associated edema; however, they may be helpful for comorbid conditions →Interventions may include the following: →Elevate limb →Compression stockings or bandages →Reduced dietary sodium intake →Optimization of volume status, consideration for diuretic treatment →Lymphedema massage of the affected area →Exercise →Recommend appropriate skin and foot care to prevent secondary cellulitis
		Grade ≥ 3	→Grade 3: withhold amivantamab until recovery to grade ≤ 1 or baseline. Resume at the same dose if recovery occurs within 1 week. Resume at a reduced dose if recovery occurs after 1 week but within 4 weeks. Permanently discontinue if recovery does not occur within 4 weeks →Grade 4: withhold amivantamab until recovery to grade ≤ 1 or baseline. Resume at a reduced dose if recovery occurs within 4 weeks. Permanently discontinue if recovery does not occur within 4 weeks and for recurrent grade 4 reactions
	Hypoalbuminemia Frequency: Ami monotherapy = 27%; Ami + Laz = 48%; Ami + CT = 22%-41%	Grades 1-2Grade 3	→Consider a nutrition consultation; however, this may have limited effect →Treat inflammatory cause of hypoalbuminemia if known→Consider dose reduction or treatment interruption
	
Other	IRRs Frequency: IV Ami monotherapy = 66%; IV Ami + Laz = 63%; IV Ami + CT = 42%-58%	→Administer prophylactic premedications (antihistamines, antipyretics, systemic corticosteroids) →Administer oral dexamethasone 8 mg BID for 2 days prior, and 1 dose 1 hour prior to amivantamab infusion on cycle 1 day 1 →Split amivantamab dose as per prescribing information Further guidance is in Figure 3	
	Stomatitis Frequency: Ami monotherapy = 21%; Ami + Laz = 29%; Ami + CT = 25%-32%	Grades 1-2	→Routine dentist appointments →Diligent oral hygiene with nonalcohol-based mouthwashes/rinses, gentle toothpaste, and soft toothbrushes →Salt or baking soda mouth rinses (1 quart water with 1 teaspoon salt and/or baking soda) up to 4 times a day →Consider topical or systemic antimicrobial agents as needed for fungal, viral, and/or bacterial infections →Consider topical corticosteroids, such as dexamethasone elixir as a mouthwash (0.5 mg per 5 mL; use 10 mL to swish and spit 4 times per day) →Consider intralesional steroids (dental or oral medicine referral)
		Grade ≥ 3	→Grade 3: withhold amivantamab until recovery to grade ≤ 1 or baseline. Resume at the same dose if recovery occurs within 1 week. Resume at a reduced dose if recovery occurs after 1 week but within 4 weeks. Permanently discontinue if recovery does not occur within 4 weeks →Grade 4: withhold amivantamab until recovery to grade ≤ 1 or baseline. Resume at a reduced dose if recovery occurs within 4 weeks. Permanently discontinue if recovery does not occur within 4 weeks and for recurrent grade 4 reactions →Consider hospitalization for supportive care if necessary
	Diarrhea Frequency: Ami monotherapy = 12%; Ami + Laz = 29%; Ami + CT = 14%-21%	Grades 1-2	→Standard antidiarrheal therapies (loperamide 4 mg PO after first loose stool, 2 mg thereafter, and not to exceed 16 mg/day followed by diphenoxylate-atropine and opium tincture if AE persists) →Attention to electrolyte replacement and hydration →Consider stool cultures
		Grade ≥ 3	→Interruption of anticancer therapy →Intensified supportive care as needed to manage electrolytes and volume depletion →Consider further diagnostics and/or GI consultation for persistent or severe diarrhea

Recommending organization: ^a^MASCC Skin Toxicity Study Group 2011; ^b^UK Expert Consensus 2015; ^c^Taiwanese Dermatological Association 2017; ^d^Spanish Academy of Dermatology and Venereology and the Spanish Society of Medical Oncology 2019; ^e^Brazil Expert Panel 2020; ^f^Oncology Nursing Society 2020; ^g^ESMO Clinical Practice Guidelines 2021.

^h^In the absence of formal guidelines, this summary includes management techniques recommended by previous review articles for the most common *MET*-related AEs associated with amivantamab and other MET-targeting agents. Treatment recommendations are based on the authors’ clinical experience. Unless specified, cream, lotion, or ointment should be chosen based on patient factors and preference.

AE, adverse event; Ami, amivantamab; BID, twice daily; CT, chemotherapy; EGFR, epidermal growth factor receptor; ESMO, European Society for Medical Oncology; GI, gastrointestinal; IRR, infusion-related reaction; IV, intravenous; Laz, lazertinib; MASCC, Multinational Association for Supportive Care in Cancer; MET, mesenchymal epithelial transition; ONS, Oncology Nursing Society; PO, orally; QD, once daily.

### Future preventive strategies

With the aim of preventing dermatologic AEs during amivantamab treatment, the phase 2 COCOON study (ClinicalTrials.gov Identifier: NCT06120140) is evaluating enhanced preventive dermatologic management in patients treated with first-line amivantamab and lazertinib. The COCOON dermatologic management regimen consists of oral doxycycline or minocycline for 12 weeks followed by topical clindamycin lotion on the scalp, chlorhexidine on the nails, and a ceramide-based moisturizer on the body and face. The first interim analysis showed that during the first 12 weeks of amivantamab plus lazertinib treatment, the incidence of grade 2 or higher dermatologic AEs was 38.6% in patients who were randomized to receive the COCOON prophylactic regimen compared with 76.5% in patients who received standard-of-care dermatologic management (OR, 0.19; 95% CI: 0.09-0.40; *P* < .0001). Substantial reductions in dermatologic AEs were observed on different body locations, including a 65% reduction in face/body dermatologic AEs (23% vs 62%), a 70% reduction in scalp AEs (9% vs 29%), and a 25% reduction in paronychia (16% vs 21%).^82^

## Incidence and management of *MET*-associated amivantamab AEs

### Common AEs

#### Peripheral edema

Peripheral edema is commonly reported with MET inhibitors in 21%-50% of patients treated with capmatinib, 51% with crizotinib, 26%-63% with tepotinib, 21%-54% with savolitinib, and 18% with amivantamab.^40,43,83^

#### Hypoalbuminemia

Hypoalbuminemia, reported in 16% of patients treated with tepotinib, 23% with savolitinib, and 27% with amivantamab monotherapy,^40,43^ is a class effect of MET inhibition.^74^ It is also a marker of inflammation and an important prognostic factor in advanced NSCLC, as declining albumin levels can occur in the setting of increased active disease burden.^75,76^

#### Other MET-related AEs

Of note, some *MET*-related AEs, such as pleural effusion and increased creatinine, have not been reported with amivantamab.^43^ Pleural effusion is reported in 8% of patients treated with tepotinib,^77^ and increased creatinine is reported in 19% of patients treated with capmatinib and 18% of patients treated with tepotinib.^43^ Although the cause of *MET*-associated AEs is not clearly identified, some may occur due to the role of MET in regulating vascular endothelial permeability^43^; thus, vascular endothelial growth factor inhibitors are currently being investigated.

In the absence of formal guidelines, Table 1 summarizes the management techniques recommended by previous review articles for the most common *MET*-related AEs associated with amivantamab and other MET-targeting agents.

### Prevention and management of peripheral edema

In addition to the strategies listed in Table 1, it is important to identify any other medical conditions (cardiac, hepatic, or renal disease) or medications (including chemotherapies, antihypertensives, and nonsteroidal anti-inflammatories) that may contribute to peripheral edema.^43^ If peripheral edema occurs, thorough examination and history taking are essential to elucidate underlying causes.^78^ Interventions other than dose interruption or modification are rarely effective.^83^

### Prevention and management of hypoalbuminemia

Prevention strategies are not commonly used for hypoalbuminemia. HCPs may refer patients to a nutritionist, and any underlying inflammatory cause should be treated (Table 1).^43,79^

## Preventive strategies for IRRs

IRRs are a common but transient AE associated with many cancer drugs administered via infusion and are characterized by symptoms such as chills, nausea, shortness of breath, hypotension, and rash.^47,48^ IRRs observed with amivantamab administration differ from the IgE-mediated allergic reactions reported with cytotoxic drugs and cytokine-release reactions seen with some other monoclonal antibodies in that they primarily occur on the first infusion and not with subsequent treatments.^48^

### Rates of IRRs with intravenous amivantamab

IRRs were reported by 66% of patients treated with IV amivantamab monotherapy, almost exclusively on days 1 or 2 of cycle 1, and at very low rates after cycle 1 day 2.^40^ In clinical trials of combination regimens, IRRs were reported in 63%-66% of patients receiving amivantamab plus lazertinib^21,58^ and 42%-58% of those receiving amivantamab plus chemotherapy.^28,32^ Most IRRs were mild to moderate, with grade ≥3 IRRs reported in ≤6% of patients across studies.^21,28,32,40^

### Preventive strategies for IRRs

Based on learnings from the phase 1 CHRYSALIS trial, the risk of IRRs with IV amivantamab has been mitigated by splitting the first dose over 2 days, reducing initial infusion rates, and administering prophylactic premedication, such as antihistamines, antipyretics, and systemic corticosteroids.^48^

The SKIPPirr study (ClinicalTrials.gov Identifier: NCT05663866) investigated the impact of oral dexamethasone administration on the incidence of IRRs following IV amivantamab.^84^ Prophylactic treatment with oral dexamethasone 8 mg twice daily on the 2 days prior to the cycle 1 day 1 infusion and 1 hour prior to infusion on cycle 1 day 1 (5 doses total) resulted in a 3-fold reduction in IRR incidence (from 67.4% to 22.5%) compared with standard IRR management.^47^ Based on these preliminary findings, this premedication regimen is recommended for primary prophylaxis against IRRs in clinical practice.

**Figure 3.**
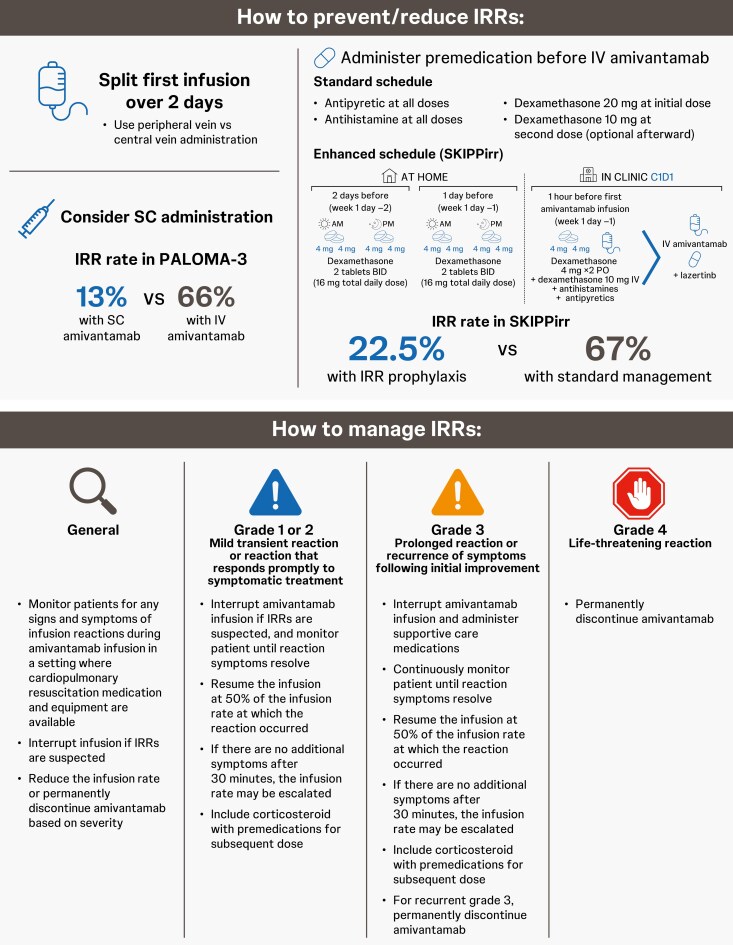
Recommended preventive and reactive strategies for IRRs.^14,47,80,81^ As of March 2025, SC amivantamab is not approved by regulatory authorities. For re-escalation of amivantamab following IRRs, please refer to the amivantamab prescribing information for more details. BID, twice daily; IRR, infusion-related reaction; IV, intravenous; PO, orally; SC, subcutaneous.

If they occur, IRRs can be managed by holding infusion, changing the amivantamab infusion flow rate, and by administration of appropriate supportive care medications (Figure 3, Table 1). Patients should be educated on IRR symptoms and instructed to tell clinic staff as soon as they are experienced; clinic staff should also carefully monitor patients during the first 1-2 hours of infusion on day 1 for any signs of IRRs.^48^

### Subcutaneous amivantamab

The PALOMA studies of SC amivantamab demonstrated a lower rate of IRRs than seen with IV amivantamab.^21,85-87^ The phase 3 randomized PALOMA-3 study evaluated SC versus IV amivantamab plus oral lazertinib in patients with *EGFR*m NSCLC after progression on both osimertinib and platinum-based chemotherapy.^80^ The rate of IRRs in 206 patients receiving SC amivantamab was about 5-fold lower than in the 210 patients receiving IV amivantamab (13% vs 66%); there were no discontinuations due to IRRs in the SC group versus 4 (2%) discontinuations in the IV group. The rates of other AEs, including dermatologic and MET-associated toxicities, observed with SC amivantamab were consistent with those associated with IV administration, reflecting systemic on-target activity.^80^ Thus, should SC amivantamab receive approval by regulatory authorities, it has the potential to reduce IRRs while maintaining efficacy.

### Other AEs

#### Common AEs

Elevations of liver enzymes, including alanine aminotransferase (ALT), aspartate aminotransferase (AST), and alkaline phosphatase, are commonly seen with anticancer therapies, including EGFR- and MET-targeted therapies, with most events being of a low grade and reversible.^43,44,88,89^ Elevations in ALT/AST with amivantamab monotherapy and combination regimens have been transient and reversible.

Gastrointestinal AEs, particularly diarrhea, are common with many classes of anticancer therapy, including EGFR and MET inhibitors, and have a substantial impact on quality of life.^43,44,90^ Diarrhea was reported in up to 95% of patients on first- and second-generation EGFR TKIs and in up to 58% of patients on osimertinib.^44^ In amivantamab trials, diarrhea was reported in 12% of patients receiving amivantamab monotherapy, 14%-21% receiving amivantamab plus chemotherapy, and 29% receiving amivantamab plus lazertinib.^21,28,32,40^

Stomatitis (or oral mucositis), caused by inflammation of the oral tissues, is a common AE with EGFR-targeted treatments.^44^ Stomatitis occurred in 21% of patients receiving amivantamab monotherapy, 29% receiving amivantamab plus lazertinib, and 25%-32% receiving amivantamab plus chemotherapy.^21,28,32,40^

#### Management of elevated liver enzymes

Regular monitoring of serum ALT and AST levels is recommended during EGFR and MET inhibitor treatment.^43,44^ Most events are low grade and reversible; however, if a drug-induced liver injury develops, the suspected causative medication should be discontinued, as there are currently no specific treatments available for drug-induced liver injury.^44^ Evaluation for alternative causes of abnormal liver enzymes and new medication or supplement exposures should also be considered.

### Prevention and management of diarrhea

The risk of diarrhea may be reduced by following a diet with low fiber and fat content and minimizing intake of fruit, red meat, alcohol, and caffeine.^43,44,64^ Diarrhea can usually be managed effectively with standard antidiarrheal therapies (Table 1).^43,44,64^

### Prevention and management of stomatitis

To reduce the risk of stomatitis, preventive measures include good oral hygiene, use of a non-alcohol mouthwash, and a diet consisting of soft, moist, non-irritating foods. Patients should be aware of the need to alert an HCP at the first signs of stomatitis.^44,64^ Management strategies are summarized in Table 1.

### AEs associated with amivantamab plus lazertinib

The combination of amivantamab with lazertinib aims to provide a synergistic treatment benefit by simultaneously targeting the extracellular and catalytic EGFR domains.^28,91^

### Common AEs

**Figure 4.**
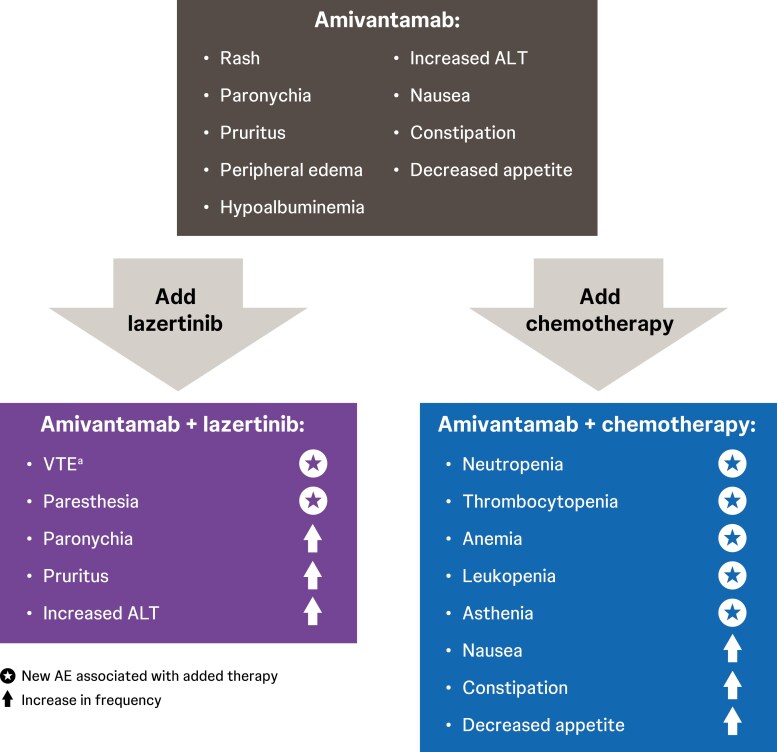
AEs associated with amivantamab combination regimens.^28,40,44,58^ AE, adverse event; ALT, alanine aminotransferase; VTE, venous thromboembolism. ^a^When initiating treatment with amivantamab in combination with lazertinib, administration of anticoagulant prophylaxis is recommended for the first 4 months of treatment to prevent VTE events (see Figure 5 for details of recommended anticoagulant regimens).^14^ Note: Management of chemotherapy-associated AEs is outside the scope of this review article.

Most AEs seen with amivantamab plus lazertinib have been consistent with the tolerability profile seen with amivantamab monotherapy.^40,58^ However, exceptions include higher incidences of pruritus with combination versus monotherapy (34% vs 17%), paronychia (64% vs 45%), hypoalbuminemia (46% vs 27%), increased ALT (32% vs 15%), dizziness (21% vs 8%), and paresthesia, an AE unique to lazertinib^25,40,58^ (25% vs not reported among the most common [≥10%]; Figure 4). These higher incidences may be explained by increased drug burden with combination therapy versus monotherapy and by the simultaneous targeting of EGFR extracellular and kinase domains. Treating providers should rule out other causes for AEs, specifically dizziness. Dizziness caused by volume depletion may be managed with IV fluids, while vertigo-induced dizziness and paresthesia may require changes in amivantamab or lazertinib regimens, or referral to neurology or physical/occupational therapy.

Management strategies for pruritus, paronychia, and hypoalbuminemia are summarized in Table 1.

### Venous thromboembolism

Patients with cancer are at an increased risk of venous thromboembolism (VTE), a major cause of cancer-related mortality.^95,96^ While tissue factor is thought to play a role in lung cancer-associated VTE,^97^ risk factors such as age ≥60 years, higher Eastern Cooperative Oncology Group performance status, and treatment response appear related to VTE incidence.^98^ Increased VTEs have been observed with the combination of amivantamab plus lazertinib, with most events (62%) occurring during the first 4 months of treatment.^21,28,98^ In the MARIPOSA study, VTE events were reported in 37% of patients receiving amivantamab plus lazertinib compared with 9% of those receiving osimertinib, although the incidence of grade 4 or 5 events was low and similar in the 2 groups.^21^ In the MARIPOSA-2 study, VTE occurred in 22% of the amivantamab-lazertinib-chemotherapy group compared with 10% of the amivantamab-chemotherapy group and 5% of patients in the chemotherapy group; the frequency of Grade ≥3 VTE was 6%, 2%, and 3% in the 3 treatment groups, respectively.^28^

**Figure 5.**
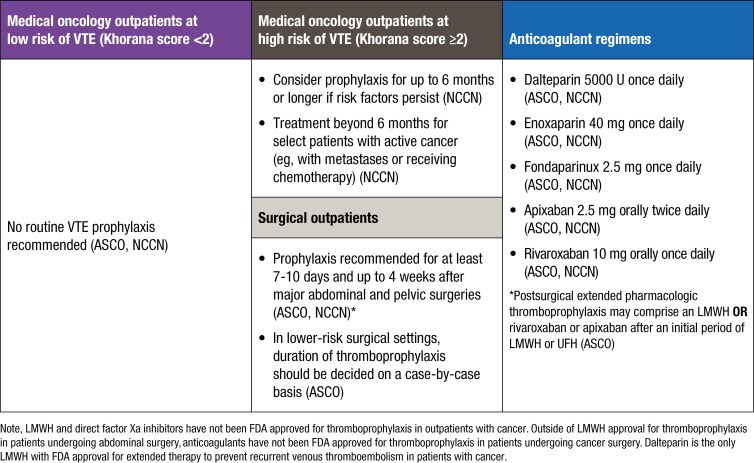
VTE prophylaxis recommendations– ASCO and NCCN Clinical Practice Guidelines in Oncology.^92–94^ ASCO, American Society of Clinical Oncology; FDA, US Food and Drug Administration; LMWH, low-molecular-weight heparin; NCCN, National Comprehensive Cancer Network; UFH, unfractionated heparin; VTE, venous thromboembolism.

Prophylactic anticoagulants are now recommended for the first 4 months of treatment with amivantamab plus lazertinib.^28,80,87,91^ Notably, VTEs were lower with SC versus IV formulations without anticoagulation, and rates were further reduced when prophylactic anticoagulation was used.^80,87^ Guideline-recommended VTE prophylaxis strategies are summarized in Figure 5 and represent reasonable options for use for anticoagulant prophylaxis during the initial months of amivantamab plus lazertinib therapy.^92,99^

### AEs associated with amivantamab combined with chemotherapy

The combination of amivantamab plus chemotherapy aims to enhance tumor response.^32^ In studies investigating amivantamab plus chemotherapy, certain AEs were more commonly observed compared with amivantamab monotherapy, including neutropenia, thrombocytopenia, anemia, leukopenia, asthenia, nausea, constipation, and decreased appetite (Figure 4).^28,40,44^ These higher incidences are likely due to both the increased drug burden of combination therapy and the addition of chemotherapy-associated AEs to those associated with EGFR and MET inhibition. All patients receiving chemotherapy-containing regimens should receive laboratory monitoring and management by a physician with experience in the management of cytotoxic chemotherapy.

Indirect comparison of AE rates in the MARIPOSA and MARIPOSA-2 studies suggests a lesser burden of dermatologic AEs with the combination of amivantamab plus chemotherapy compared with amivantamab plus lazertinib (the 2 regimens have not yet been compared in a head-to-head trial). For example, rash was reported in 43% (6% grade ≥3) of patients receiving amivantamab plus chemotherapy compared with 64% (17% grade ≥3) of patients receiving amivantamab plus lazertinib.^16,28^ Likewise, paronychia was reported in 37% of patients treated with amivantamab plus chemotherapy and 69% of patients treated with amivantamab plus lazertinib.^16,28^ The results across these 2 studies suggest that cutaneous AEs with amivantamab are exacerbated by the addition of lazertinib (or potentially EGFR-targeted therapy in general) compared with chemotherapy agents with non-overlapping AE profiles.

### Summary, conclusions, and future directions

The introduction of amivantamab to the treatment options for advanced NSCLC has provided improved clinical outcomes compared with previously available therapies. As an EGFR- and MET-targeting therapy, amivantamab is associated with various AEs, including skin reactions, peripheral edema, hypoalbuminemia, IRRs, gastrointestinal AEs, and stomatitis. While some AEs appear to be a result of MET inhibition, MET inhibition provides a key benefit of amivantamab treatment in overcoming common resistance mechanisms.^21,28^ Combination regimens of amivantamab plus lazertinib and/or chemotherapy have been associated with additional AEs, such as VTEs or hematologic AEs, respectively.^21,58^

**Figure 6.**
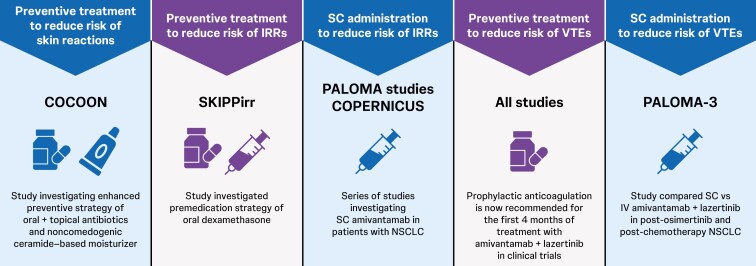
Current research investigating preventive strategies for amivantamab AEs.^68,84,80,85,86,100,101^ AE, adverse event; IRR, infusion-related reaction; IV, intravenous; NSCLC, non-small cell lung cancer; SC, subcutaneous; VTE, venous thromboembolism. ClinicalTrials.gov ID: COCOON, NCT06120140; SKIPPirr: NCT05663866; COPERNICUS, NCT06667076; PALOMA, NCT04606381; PALOMA-3, NCT05388669

Several novel strategies are under investigation to mitigate AEs associated with amivantamab treatment (Figure 6). These include enhanced prophylactic dermatologic regimens to reduce the risk of cutaneous toxicity as evaluated within the COCOON study,^82^ enhanced steroid premedication to reduce the risk of infusion reactions,^84^ and addition of anticoagulation prophylaxis to reduce the risk of VTE.^80,87^ Potential SC administration, should this become available outside of a clinical trial setting, may further mitigate the risk of infusion reactions and VTEs, while maintaining efficacy and improving logistical feasibility and convenience.^80,85-87^ The ongoing COPERNICUS study (NCT06667076) is evaluating treatment outcomes in the context of these optimized supportive care regimens using SC amivantamab every 4 weeks.^100^

These AE management options and a better understanding of anticipated AEs with amivantamab regimens may help improve the patient experience and reduce the need for treatment interruption, resulting in improved treatment outcomes.

## Data Availability

The data sharing policy of Janssen Pharmaceutical Companies of Johnson & Johnson is available at https://innovativemedicine.jnj.com/our-innovation/clinical-trials/transparency
